# Supplementary material for: Opening a can of worms: a test of the co-infection facilitation hypothesis
Source: Oecologia. 2023 Jun 30;204(2):317–25. doi: 10.1007/s00442-023-05409-7 (PMC10756930; doi:10.1007/s00442-023-05409-7)
Supplement: Supplementary file 1 [file 442_2023_5409_MOESM1_ESM.docx]

**SUPPLEMENTARY INFORMATION**

**Opening a can of worms: a test of the coinfection facilitation hypothesis**

Maria L. Rodgers^1,2^, Daniel I. Bolnick^1^

^1^Department of Ecology and Evolutionary Biology, University of Connecticut, Storrs CT 06269, USA

Current affiliation: ^2^Department of Biological Sciences, North Carolina State University, Morehead City, NC 28557, USA

**Fig. S1.** Density plot of the distribution of (A) the raw parasite richness for all individual fish in the dataset, with versus without *S. solidus* infection, and (B) residual parasite richness using a whole-dataset Poisson GLM to control for effects of population (random effect), log length, and sex. The latter metric is shown in Fig. 1C, which focuses on the mean and confidence of the two curves shown here

	

**Fig. S2.** A re-analysis similar to Figure 1, but for another focal parasite, *Crepidostomum spp.*

 

**Fig. S3.** A re-analysis similar to Figure 1, but for another focal parasite, *Diplostomum spp*

**Fig. S4.** A re-analysis similar to Figure 1, but for another focal parasite, *Bunoderina sp*. Although richness is consistently higher in infected than in *Bunoderina*-uninfected fish (top panel), this effect is driven by a joint dependence on host size. After accounting for host size, the residual parasite richness is not significantly associated with *Bunoderina* infection status (bottom panel)
